# Supplementary material for: Evaluation and comparison of nine growth and development-based measures of pubertal timing
Source: Commun Med (Lond). 2024 Aug 7;4:159. doi: 10.1038/s43856-024-00580-1 (PMC11306255; doi:10.1038/s43856-024-00580-1)

Evaluation and comparison of nine growth and development-based measures of pubertal timing

Ahmed Elhakeem[1,2] *, Monika Frysz[1,3], Ana Goncalves Soares[1,2], Joshua A Bell[1,2], Tim J Cole[4], Jon Heron[1,2], Laura D Howe[1,2], Sylvain Sebert[5], Kate Tilling[1,2], Nicholas J Timpson[1,2], Deborah A Lawlor[1,2,6]

Contents

Supplementary Table 1 Description of the repeated pubertal growth and development data and the puberty age measures derived from these 2

Supplementary Table 2. Number and age of study participants that completed each research clinic assessment and puberty questionnaire 3

Supplementary Table 3. Numbers of study participants that had help from parent/guardian with completing each puberty questionnaire 4

Supplementary Table 4. BIC values from mixed effects models with different degrees of freedom (df) 5

Supplementary Table 5. Correlations between the size, timing, intensity, and post-growth random effects from mixed effects models that included at least two of these random effects 6

Supplementary Table 6. Comparison of the included study participants with data from at least one puberty questionnaire or research clinic with those excluded due to missing data 7

Supplementary Note Instructions provided to the ALSPAC study participants for reporting Tanner stages using repeated puberty questionnaires from ages 7-17 years 8

Supplementary Figure 1 Distribution of standardized genetic risk scores........ 12

Supplementary Figure 2 Estimated mean distance and velocity curves for height, weight, and BMC from the selected mixed effects models 13

Supplementary Figure 3 Estimated mean distance and velocity curves for Tanner stages, axillary hair, and voice break from the selected mixed effects models 14

Supplementary Table 1 Description of the repeated pubertal growth and development data and the puberty age measures derived from these

Data type (number of repeated measures)	Description and source of the prospective repeated puberty data collections	Definition of the derived measures of pubertal age (months), method
Height (nine repeated measures)	Measured to the nearest 0.1 cm using a Harpenden stadiometer during nine research clinics from mean age 7.6 to 17.8 years by accredited fieldworkers	age at peak height velocity, mixed effects model
Weight (nine repeated measures)	Measured to the nearest 0.1 kg using a Tanita Body Fat Analyser during nine research clinics from mean age 7.6 to 17.8 years by accredited fieldworkers	age at peak weight velocity, mixed effects model
Bone mass (five repeated measures)	Measured by Lunar Prodigy Dual-energy X-ray Absorptiometry (DXA) scans as total body (less head) bone mineral content (BMC; grams) during five research clinics from mean age 9.9 to 17.8 years by accredited fieldworkers	age at peak BMC velocity, mixed effects model
Tanner pubic hair development stage (nine repeated measures)	Reported by parent and/or child during nine puberty questionnaires from mean age 8.2 to 17.0 years, by using line drawings with accompanying descriptions of the five Tanner stages of pubic hair development to identify the stage most closely matched to the child's current stage.	age in Tanner stage 3 of pubic hair development, mixed effects model
Tanner breast development stage (nine repeated measures)	Reported by parent and/or child during nine puberty questionnaires from mean age 8.2 to 17.0 years, by using line drawings with accompanying descriptions of the five Tanner stages of breast development to identify the stage most closely matched to the child's current stage.	age in Tanner stage 3 of breast development, mixed effects model
Tanner genitalia development stage (nine repeated measures)	Reported by parent and/or child during nine puberty questionnaires from mean age 8.2 to 17.0 years, by using line drawings with accompanying descriptions of the five Tanner stages of genitalia development to identify the stage most closely matched to the child's current stage.	age in Tanner stage 3 of genitalia development, mixed effects model
Menstruation (nine repeated measures)	Reported by parent and/or child during nine puberty questionnaires from mean age 8.2 to 17.0 years, in response to questions on whether the daughter's first menstrual period occurred and if it has, the month and year it started.	age at menarche, the first reported age at onset of menstruation
Voice change (eight repeated measures)	Reported by parent and/or child during eight puberty questionnaires from mean age 9.7 to 17.0 years, in response to a question on whether the son's voice (1) has changed and if it has, whether it was (2) occasionally a lot lower or if the voice had (3) changed totally.	age at voice break, mixed effects model
Axillary hair (seven repeated measures)	Reported by parent and/or child during seven puberty questionnaires from mean age 9.7 to 17.0 years, in response to a question on whether the child had (1) started growing hair in the armpits or (2) not yet started.	age at axillary hair, mixed effects model

Supplementary Table 2. Number and age of study participants that completed each research clinic assessment and puberty questionnaire

	Females			Males	
	N	mean age (SD)		N	mean age (SD)
Research Clinic (C): height, weight, and bone mineral content (BMC) assessments					
C1 (height, weight)	4,047	7.6 (0.3)		4,170	7.6 (0.3)
C2 (height, weight)	3,576	8.7 (0.3)		3,598	8.7 (0.3)
C3 (height, weight, BMC)	3,865	9.9 (0.3)		3,772	9.9 (0.3)
C4 (height, weight)	3,785	10.7 (0.3)		3,699	10.7 (0.3)
C5 (height, weight, BMC)	3,611	11.8 (0.2)		3,496	11.8 (0.2)
C6 (height, weight)	3,455	12.8 (0.2)		3,320	12.9 (0.2)
C7 (height, weight, BMC)	3,118	13.9 (0.2)		3,009	13.9 (0.2)
C8 (height, weight, BMC)	2,856	15.5 (0.4)		2,572	15.5 (0.3)
C9 (height, weight, BMC)	2,851	17.8 (0.5)		2,217	17.8 (0.4)
N with ≥1 height assessment	4,186	-		4,251	-
N with ≥1 weight assessment	4,183	-		4,248	-
N with ≥1 BMC assessment	3,747	-		3,680	-
Puberty Questionnaire (Q): Tanner pubic hair, breast, and genitalia stages, and axillary hair, voice break, and menarche data collection					
Q1 (no axillary hair or voice break)	3,298	8.2 (0.3)		2,947	8.2 (0.3)
Q2 (no axillary hair)	3,652	9.7 (0.1)		3,357	9.7 (0.1)
Q3 (no axillary hair)	3,482	10.7 (0.1)		3,156	10.7 (0.1)
Q4 (all)	3,331	11.7 (0.1)		2,991	11.7 (0.1)
Q5 (all)	3,195	13.1 (0.2)		2,871	13.1 (0.2)
Q6 (all)	2,868	14.7 (0.1)		2,286	14.7 (0.1)
Q7 (all)	2,565	15.4 (0.3)		2,292	15.4 (0.3)
Q8 (all)	2,813	16.1 (0.1)		1,938	16.1 (0.1)
Q9 (all)	2,600	17.0 (0.1)		1,762	17.0 (0.1)
N with ≥1 Tanner pubic hair response	4,276	-		4,074	-
N with ≥1 Tanner genitalia response	-	-		4,041	-
N with ≥1 Tanner breasts response	4,273	-		-	-
N with ≥1 axillary hair response	4,031	-		3,804	-
N with ≥1 voice break response	-	-		4,020	-
N with data on age at menarche	3,457	-		-	-

Supplementary Table 3. Numbers of study participants that had help from parent/guardian with completing each puberty questionnaire

	Females			Males	
	N	%		N	%
Child had help completing puberty questionnaire (Q)					
Q1					
Yes	3241	99.0		2871	98.4
No	33	1.0		48	1.6
Q2					
Yes	3544	97.7		3107	96.5
No	82	2.3		114	3.5
Q3					
Yes	3379	97.8		2874	94.9
No	75	2.2		153	5.1
Q4					
Yes	2903	87.8		2311	80.9
No	404	12.2		545	19.1
Q5					
Yes	2500	79.2		1975	71.8
No	656	20.8		774	28.2
Q6					
Yes	404	14.3		246	11.0
No	2419	85.7		1988	89.0
Q7					
Yes	319	12.7		142	6.4
No	2199	87.3		2071	93.6
Q8					
Yes	250	9.0		141	7.4
No	2532	91.0		1769	92.6
Q9					
Yes	146	5.7		93	5.4
No	2427	94.3		1644	94.6

Supplementary Table 4. BIC values from mixed effects models with different degrees of freedom (df)

	BIC			
	df=3 (2 knots)	df=4 (3 knots)	df=5 (4 knots)	df=6 (5 knots)
Females				
Height	114776	114369	114218	**114132**
Weight	143063	143068	**142163**	142746
BMC	**165547**	167436	nc	170841
Tanner pubic hair stage	38159	**37177**	37531	36911
Tanner breast stage	**40363**	nc	40783	40640
Axillary hair	-10348	-12226	-20485	**-27678**
Males				
Height	nc	116185	115770	**115730**
Weight	138753	nc	**134206**	nc
BMC	151301	nc	**152763**	154855
Tanner pubic hair stage	29076	29182	28998	29379
Tanner genitalia stage	**44390**	44812	45152	46142
Axillary hair	-530	-4982	**-9733**	-8740
Voice break	15258	14995	14683	**14619**

nc=model did not converge. Selected models are highlighted in bold font.

Supplementary Table 5. Correlations between the size, timing, intensity, and post-growth random effects from mixed effects models that included at least two of these random effects

	Correlations between random effects					
	timing and intensity	timing and size	size and intensity	timing and post-growth	size and post-growth	intensity and post-growth
Females						
Height	0.07	0.30	0.43	-	-	-
Weight	0.26	0.18	0.42	-0.04	0.78	-0.16
BMC	0.37	0.19	0.68	-0.63	0.53	-0.63
Tanner pubic hair stage	-0.50	-	-	-	-	-
Tanner breast stage	0.19	-	-	-	-	-
Males						
Height	0.15	0.37	0.56			
Weight	0.75	0.49	0.55	-0.11	0.68	-0.15
BMC	0.21	0.37	0.56	0.35	0.93	0.23
Tanner pubic hair stage	0.66	-	-	-	-	-

Age modelled as log(age) for all measures (except BMC in females)

Supplementary Table 6. Comparison of the included study participants with data from at least one puberty questionnaire or research clinic with those excluded due to missing data

	Males		Females	
	Included (4,251)	Excluded (1,260)	Included (4,276)	Excluded (975)
Maternal pregnancy smoking [N (%)]				
No	3729 (95.8)	408 (90.7)	3750 (94.9)	278 (93.3)
Yes	164 (4.2)	42 (9.3)	200 (5.1)	20 (6.7)
Maternal education [N (%)]				
CSE	588 (14.4)	158 (29.8)	596 (14.6)	106 (30.1)
Vocational	380 (9.3)	73 (13.8)	378 (9.3)	37 (10.5)
O level	1467 (35.8)	183 (34.5)	1429 (35.0)	126 (35.8)
A level	1054 (25.7)	81 (15.3)	1037 (26.4)	57 (16.2)
Degree	608 (14.8)	35 (6.6)	641 (15.7)	26 (7.4)
Maternal parity [N (%)]				
0	1808 (45.2)	201 (39.7)	1820 (45.7)	138 (41.1)
1	1425 (35.6)	187 (37.0)	1469 (36.9)	111 (33.0)
2	572 (14.3)	77 (15.2)	526 (13.2)	60 (17.9)
3 or more	198 (5.0)	41 (8.1)	170 (4.3)	27 (8.0)
Maternal pregnancy BMI – kg/m^2 [mean (SD)]	23.0 (3.8)	22.9 (3.8)	22.9 (3.8)	23.0 (4.2)
Maternal age at birth – years [mean (SD)]	29.1 (4.6)	27.0 (4.9)	28.9 (4.5)	27.1 (4.7)
Child energy intake – kJ/day [mean (SD)]	7780 (1832)	7901 (2011)	7516 (1727)	8277 (2068)

Supplementary Note Instructions provided to the ALSPAC study participants for reporting Tanner stages using repeated puberty questionnaires from ages 7-17 years

The drawings below show stages of the way the **breasts** develop. A teenager can go through each of the five stages shown, although some teenagers skip some stages. **Please look at each of the drawings.** It is also important to read the descriptions.

Cross the box that is **closest** to your current breast stage

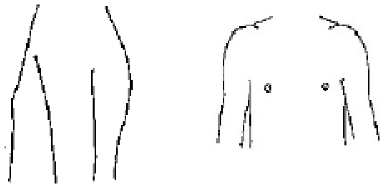

1 ☐

The nipple is raised a little in this stage. The rest of the breast is still flat.

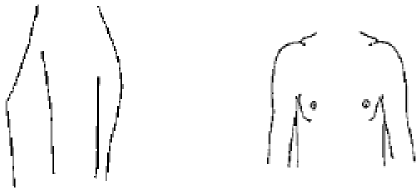

2 ☐

This is the breast bud stage. In this stage the nipple is raised more than in stage 1. The breast is a small mound. The dark area around the nipple (areola) is larger than in stage 1.

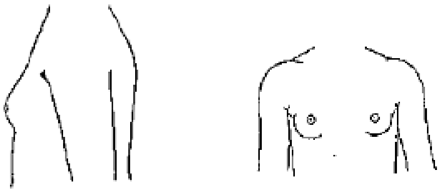

3 ☐

The areola and the breast are both larger than in stage 2. The areola does not stick out away from the breast

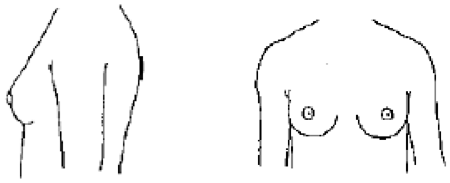

4 ☐

The areola and the nipple make up a mound that sticks up above the shape of the breast. (Note: This stage may not happen at all for some teenagers. Some teenagers develop from stage 3 to stage 5 with no stage 4.)

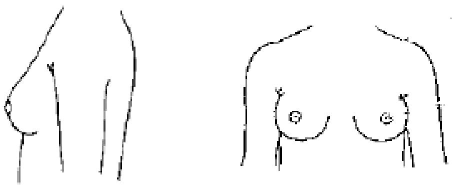

5 ☐

This is the mature adult stage. The breasts are fully developed. Only the nipple sticks out in this stage. The areola has moved back in the general shape of the breast.

The drawings below show different amounts of **female pubic hair**. A teenager can go through each of the five stages shown. **Please look at each of the drawings**. It is also important to read the descriptions.

Cross the box that is the **closest** to the amount of pubic hair you have.

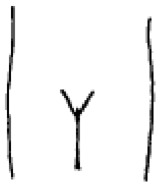

1 ☐ There is no pubic hair

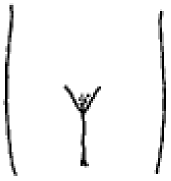

2 ☐ There is a little long, lightly coloured hair. This hair may be straight or a little curly.

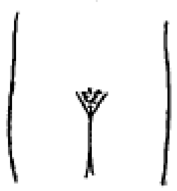

3 ☐ The hair is darker in this stage. It is coarser and more curled. It has spread out and thinly covers a bigger area.

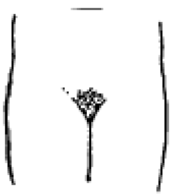

4 ☐ The hair is now as dark, curly, and coarse as that of an adult woman. However, the area that the hair covers is not as large as that of an adult woman. The hair has not spread out to the legs.

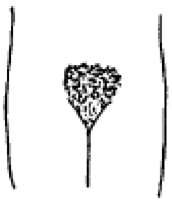

5 ☐ The hair now is like that of an adult woman. It also covers the same area as that of an adult woman. The hair usually forms a triangular pattern as it spreads out to the legs.

NOTE: Your pubic hair stage may or may not be the same as your stage of breast development.

38452

Teenagers go through the various stages of physical development at different ages.
Some start as early as 6, others not until they are 20.
We need your help in letting us know what stage you are at.
Please look at each of the drawings. It is also important to read the descriptions.

Cross the **box** that is **closest to your current stage**

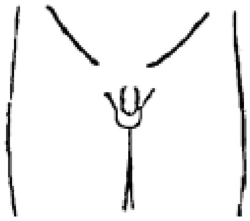

1 ☐ The size and shape of the testes, scrotum (the sac holding the testes) and penis are about the same as when you were younger.

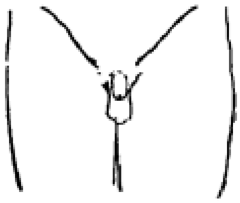

2 ☐ The penis is a little bit bigger. The scrotum has dropped and the skin of the scrotum has changed. The testes are bigger.

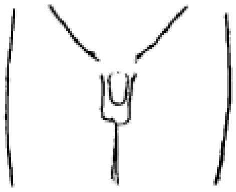

3 ☐ The penis has grown longer, the testes have grown and dropped lower.

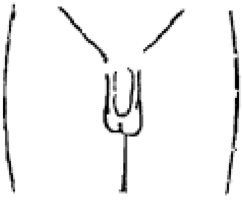

4 ☐ The penis is longer and wider. The head of the penis is bigger, the scrotum is a darker colour and bigger. The testes are bigger.

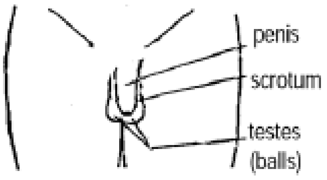

5 ☐ The penis, scrotum and testes are the size and shape of a man's.

As part of development, at some stage hair will start to grow just above the penis

Please look at each of the drawings. It is also important to read the description

Cross the box that is **closest** to the amount of pubic hair that you have.

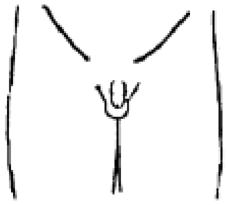

1 ☐ There is no hair at all.

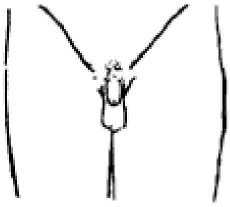

2 ☐ There is a little soft, long, lightly coloured hair at the base of the penis. It may be straight or a little curly.

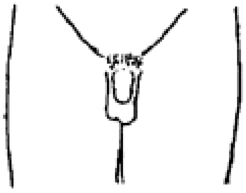

3 ☐ The hair is darker and more curled. It has spread out and thinly covers a bigger area.

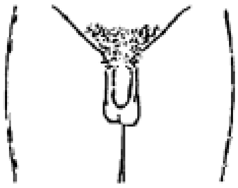

4 ☐ The hair is as dark and curly as that of a man, but it hasn't spread out to the legs.

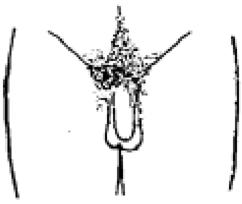

5 ☐ The hair is like that of a man. It has spread out to the legs.

Figures reproduced with consent from the ALSPAC Executive

Supplementary Figure 1 Distribution of standardized genetic risk scores

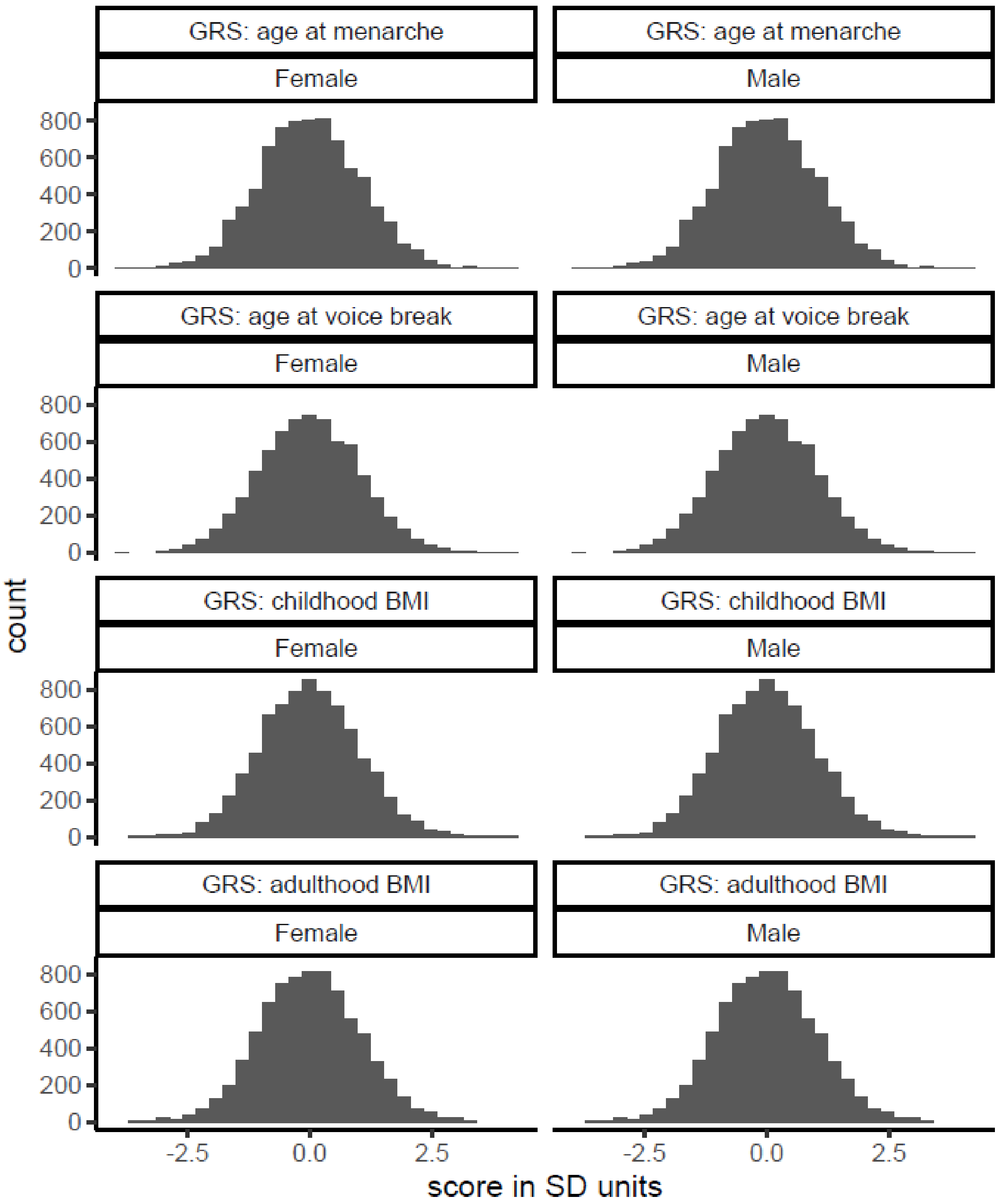

Supplementary Figure 2 Estimated mean distance and velocity curves for height, weight, and BMC from the selected mixed effects models

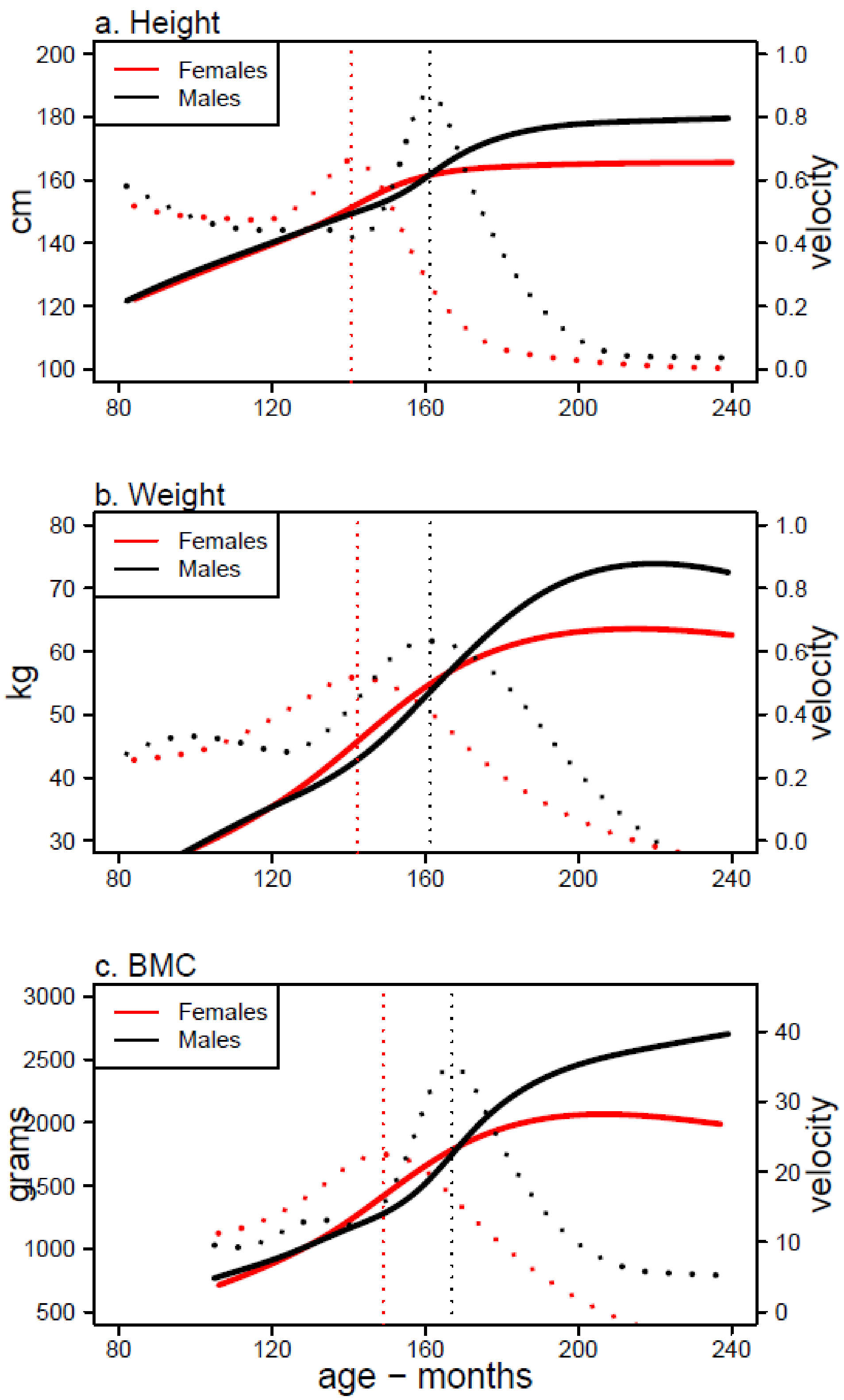

Supplementary Figure 3 Estimated mean distance and velocity curves for Tanner stages, axillary hair, and voice break from the selected mixed effects models

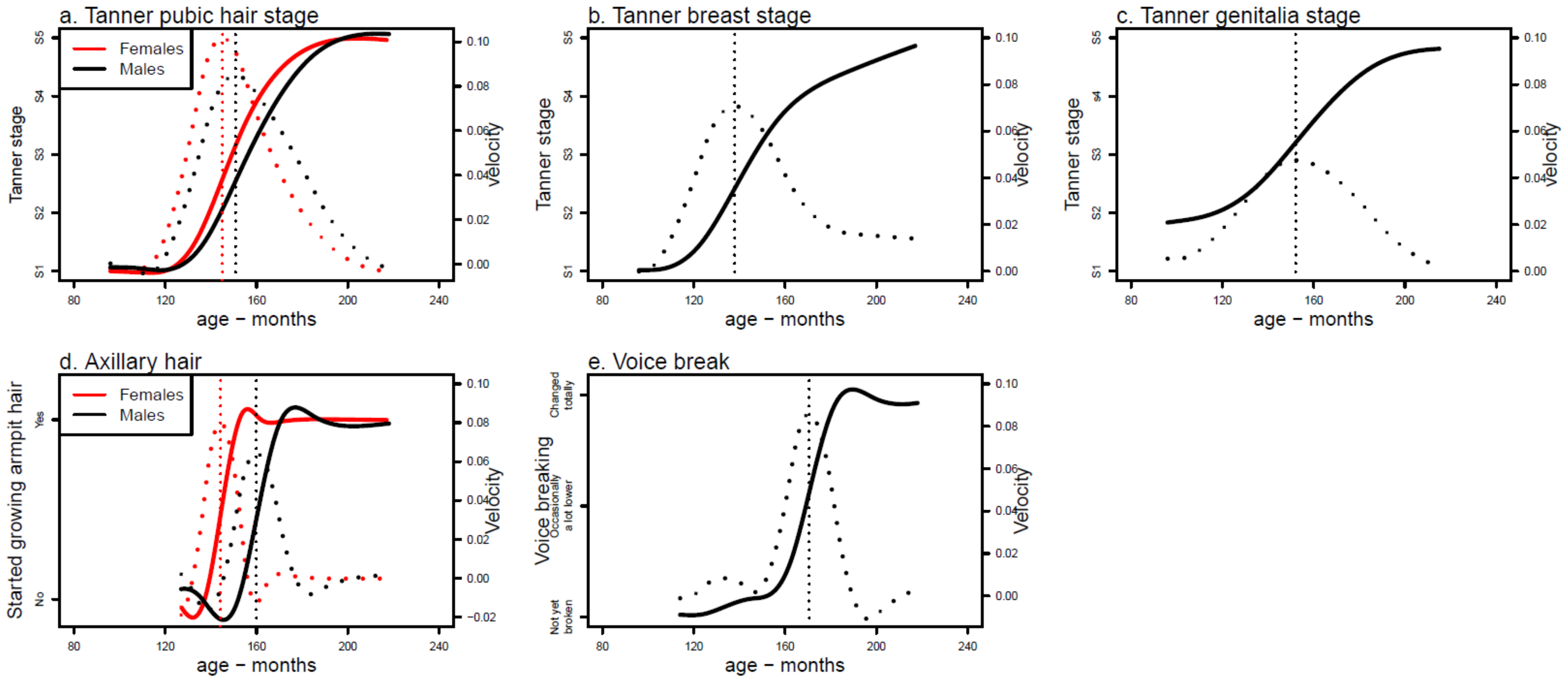

Supplement: Supplementary file 2 — Supplementary Information. [file 43856_2024_580_MOESM2_ESM.pdf]
