# Supplementary material for: Evaluation and comparison of nine growth and development-based measures of pubertal timing
Source: Commun Med (Lond). 2024 Aug 7;4:159. doi: 10.1038/s43856-024-00580-1 (PMC11306255; doi:10.1038/s43856-024-00580-1)
Supplement: Supplementary file 3 — Description of Additional Supplementary Files [file 43856_2024_580_MOESM3_ESM.pdf]

Description of Additional Supplementary Files

File name- Supplementary Data 1

File description- Genetic correlations were estimated using LD score regression with summary GWAS data obtained from Day et al. for age at menarche and in ALSPAC for all other measures of pubertal age. Note, genetic correlation estimates are unbounded, so some results fall outside 1. If the true genetic correlation is near 1, and the estimated genetic correlation is the true value plus or minus some estimation error, it's possible that the estimate (true genetic correlation + error) will be greater than 1. For many traits there was low sample size which can lead to unstable results (i.e., more likely to estimate genetic correlation > 1). When both the true genetic correlation and sample overlap are high, LD score regression can give exaggerated estimates (>1).

File name- Supplementary Data 2

File description- Source data underlying the graphs presented in figures 1-6.
