# Supplementary material for: Evaluation and comparison of nine growth and development-based measures of pubertal timing
Source: Commun Med (Lond). 2024 Aug 7;4:159. doi: 10.1038/s43856-024-00580-1 (PMC11306255; doi:10.1038/s43856-024-00580-1)
Supplement: Supplementary file 6 — Reporting Summary [file 43856_2024_580_MOESM6_ESM.pdf]

nature portfolio

Corresponding author(s): Ahmed Elhakeem

Last updated by author(s): Jun 25, 2025

Reporting Summary

Nature Portfolio wishes to improve the reproducibility of the work that we publish. This form provides structure for consistency and transparency in reporting. For further information on Nature Portfolio policies, see our Editorial Policies and the Editorial Policy Checklist.

Statistics

For all statistical analyses, confirm that the following items are present in the figure legend, table legend, main text, or Methods section.

n/a	Confirmed	
☐	☒	The exact sample size (*n*) for each experimental group/condition, given as a discrete number and unit of measurement
☐	☒	A statement on whether measurements were taken from distinct samples or whether the same sample was measured repeatedly
☐	☒	The statistical test(s) used AND whether they are one- or two-sided<br>*Only common tests should be described solely by name; describe more complex techniques in the Methods section.*
☐	☒	A description of all covariates tested
☐	☒	A description of any assumptions or corrections, such as tests of normality and adjustment for multiple comparisons
☐	☒	A full description of the statistical parameters including central tendency (e.g. means) or other basic estimates (e.g. regression coefficient) AND variation (e.g. standard deviation) or associated estimates of uncertainty (e.g. confidence intervals)
☐	☒	For null hypothesis testing, the test statistic (e.g. *F*, *t*, *r*) with confidence intervals, effect sizes, degrees of freedom and *P* value noted<br>*Give P values as exact values whenever suitable.*
☒	☐	For Bayesian analysis, information on the choice of priors and Markov chain Monte Carlo settings
☐	☒	For hierarchical and complex designs, identification of the appropriate level for tests and full reporting of outcomes
☐	☒	Estimates of effect sizes (e.g. Cohen's *d*, Pearson's *r*), indicating how they were calculated

Our web collection on statistics for biologists contains articles on many of the points above.

Software and code

Policy information about availability of computer code

Data collection	no software was used (this was a secondary analysis of cohort study data)
Data analysis	All other analyses were performed in R version 4.02 (R Project for Statistical Computing).

For manuscripts utilizing custom algorithms or software that are central to the research but not yet described in published literature, software must be made available to editors and reviewers. We strongly encourage code deposition in a community repository (e.g. GitHub). See the Nature Portfolio guidelines for submitting code & software for further information.

Data

Policy information about availability of data

All manuscripts must include a data availability statement. This statement should provide the following information, where applicable:

- Accession codes, unique identifiers, or web links for publicly available datasets
- A description of any restrictions on data availability
- For clinical datasets or third party data, please ensure that the statement adheres to our policy

Researchers interested in accessing ALSPAC data used in this study will need to submit a research proposal (https://proposals.epi.bristol.ac.uk/) for consideration by the ALSPAC Executive Committee (managed access).

Research involving human participants, their data, or biological material

Policy information about studies with human participants or human data. See also policy information about sex, gender (identity/presentation), and sexual orientation and race, ethnicity and racism.

Reporting on sex and gender	Sex-based analyses were performed
Reporting on race, ethnicity, or other socially relevant groupings	Association of childhood body composition with pubertal age measures was adjusted for possible confounders: maternal age at birth, maternal education, parity, maternal early pregnancy BMI, maternal pregnancy smoking, and childhood dietary intake. Analysis was done in White/European individuals.
Population characteristics	The initial number of pregnancies enrolled was 14,541. Of these initial pregnancies, there was a total of 14,676 fetuses, resulting in 14,062 live births and 13,988 children who were alive at 1 year of age. When children were approximately 7 years old, an attempt was made to bolster the initial sample with eligible new cases. Total sample size for analyses using data collected after age 7 years was 15,447 pregnancies, and 15,658 offspring. Of these 14,901 were alive at 1 year of age. Detailed data have been collected from offspring and parents by questionnaires, data extraction from medical records, data linkage to health records, and dedicated clinic assessments.
Recruitment	ALSPAC is a multigenerational prospective birth cohort study that recruited pregnant women residing within the catchment area of three National Health Service authorities in southwest England with an expected date of delivery between April 1991 and December 1992
Ethics oversight	ALSPAC participants provided written informed consent or assent for all measurements. Ethical approval for the ALSPAC study was obtained from the ALSPAC Law and Ethics Committee and the Local Research Ethics Committees. Consent for biological samples has been collected in accordance with the Human Tissue Act (2004). Details of all available data can be found in the ALSPAC study website which includes a fully searchable data dictionary and variable search tool (http://www.bristol.ac.uk/alspac/researchers/our-data/).

Note that full information on the approval of the study protocol must also be provided in the manuscript.

Field-specific reporting

Please select the one below that is the best fit for your research. If you are not sure, read the appropriate sections before making your selection.

☐ Life sciences ☒ Behavioural & social sciences ☐ Ecological, evolutionary & environmental sciences

For a reference copy of the document with all sections, see nature.com/documents/nr-reporting-summary-flat.pdf

Behavioural & social sciences study design

All studies must disclose on these points even when the disclosure is negative.

Study description	cohort study
Research sample	ALSPAC is a multigenerational prospective birth cohort study that recruited pregnant women residing within the catchment area of three National Health Service authorities in southwest England with an expected date of delivery between April 1991 and December 1992
Sampling strategy	ALSPAC is a multigenerational prospective birth cohort study that recruited pregnant women residing within the catchment area of three National Health Service authorities in southwest England with an expected date of delivery between April 1991 and December 1992. Pregnant women resident in Avon, UK with expected dates of delivery between 1st April 1991 and 31st December 1992 were invited to take part in the study.
Data collection	Detailed data have been collected from offspring and parents by questionnaires, data extraction from medical records, data linkage to health records, and dedicated clinic assessments.
Timing	1990 for 25 years
Data exclusions	All analyses were restricted to White ethnicity individuals (>95% of all participants) to enable consistency across phenotypic and genetic analyses.
Non-participation	The initial number of pregnancies enrolled was 14,541. Of these initial pregnancies, there was a total of 14,676 fetuses, resulting in 14,062 live births and 13,988 children who were alive at 1 year of age. When children were approximately 7 years old, an attempt was made to bolster the initial sample with eligible new cases. Total sample size for analyses using data collected after age 7 years was 15,447 pregnancies, and 15,658 offspring. Of these 14,901 were alive at 1 year of age. Detailed data have been collected from offspring and parents by questionnaires, data extraction from medical records, data linkage to health records, and dedicated clinic assessments.
Randomization	NA - This was a cohort study

Reporting for specific materials, systems and methods

We require information from authors about some types of materials, experimental systems and methods used in many studies. Here, indicate whether each material, system or method listed is relevant to your study. If you are not sure if a list item applies to your research, read the appropriate section before selecting a response.

Materials & experimental systems

n/a	Involved in the study
☒	☐ Antibodies
☒	☐ Eukaryotic cell lines
☒	☐ Palaeontology and archaeology
☒	☐ Animals and other organisms
☒	☐ Clinical data
☒	☐ Dual use research of concern
☒	☐ Plants

Methods

n/a	Involved in the study
☒	☐ ChIP-seq
☒	☐ Flow cytometry
☒	☐ MRI-based neuroimaging

Plants

Seed stocks

Report on the source of all seed stocks or other plant material used. If applicable, state the seed stock centre and catalogue number. If plant specimens were collected from the field, describe the collection location, date and sampling procedures.

Novel plant genotypes

Describe the methods by which all novel plant genotypes were produced. This includes those generated by transgenic approaches, gene editing, chemical/radiation-based mutagenesis and hybridization. For transgenic lines, describe the transformation method, the number of independent lines analyzed and the generation upon which experiments were performed. For gene-edited lines, describe the editor used, the endogenous sequence targeted for editing, the targeting guide RNA sequence (if applicable) and how the editor was applied.

Authentication

Describe any authentication procedures for each seed stock used or novel genotype generated. Describe any experiments used to assess the effect of a mutation and, where applicable, how potential secondary effects (e.g. second site T-DNA insertions, mosiacism, off-target gene editing) were examined.
